# Characterization of a Trispecific PD-L1 Blocking Antibody That Exhibits EGFR-Conditional 4-1BB Agonist Activity

**DOI:** 10.3390/antib13020034

**Published:** 2024-04-24

**Authors:** Laura Rubio-Pérez, Susana Frago, Marta Compte, Rocío Navarro, Seandean L. Harwood, Rodrigo Lázaro-Gorines, Marina Gómez-Rosel, Oana Hangiu, Noelia Silva-Pilipich, Lucía Vanrell, Cristian Smerdou, Luis Álvarez-Vallina

**Affiliations:** 1 Cancer Immunotherapy Unit (UNICA), Department of Immunology, Hospital Universitario12 de Octubre (H12O), 28041 Madrid, Spain; laura.rubio.imas12@h12o.es (L.R.-P.); rlazaro@cnio.es (R.L.-G.); marinagomezrosel.imas12@h12o.es (M.G.-R.); oana.hangiu@leadartis.com (O.H.); 2 Immuno-Oncology and Immunotherapy Group, Instituto de Investigación Sanitaria 12 de Octubre (imas12), 28041 Madrid, Spain; 3 H12O-CNIO Cancer Immunotherapy Clinical Research Unit, Spanish National Cancer Research Centre (CNIO), 28029 Madrid, Spain; 4 Chair for Immunology UFV/Merck, Universidad Francisco de Vitoria (UFV), Pozuelo de Alarcón, 28223 Madrid, Spain; 5 Department of Antibody Engineering, Leadartis SL, QUBE Technology Park, Tres Cantos, 28760 Madrid, Spain; susana.frago@leadartis.com (S.F.); marta.compte@leadartis.com (M.C.); rocio.navarro@leadartis.com (R.N.); 6 Department of Molecular Biology and Genetics, Aarhus University, 8000 Aarhus C, Denmark; sdlh@mbg.au.dk; 7 Division of DNA and RNA Medicine, Cima Universidad de Navarra, 31008 Pamplona, Spain; nsilva.1@alumni.unav.es (N.S.-P.); csmerdou@unav.es (C.S.); 8 Instituto de Investigación Sanitaria de Navarra (IdISNA) and CCUN, 31008 Pamplona, Spain; 9 Facultad de Ingeniería, Universidad ORT Uruguay, 11100 Montevideo, Uruguay; lvanrell@unav.es; 10 Nanogrow Biotech, Montevideo 11500, Uruguay

**Keywords:** cancer immunotherapy, trispecific antibody, epithelial growth factor receptor, immune checkpoint blockade, 4-1BB costimulation

## Abstract

Immune checkpoint blockade has changed the treatment paradigm for advanced solid tumors, but the overall response rates are still limited. The combination of checkpoint blockade with anti-4-1BB antibodies to stimulate tumor-infiltrating T cells has shown anti-tumor activity in human trials. However, the further clinical development of these antibodies has been hampered by significant off-tumor toxicities. Here, we generated an anti-4-1BB/EGFR/PD-L1 trispecific antibody consisting of a triple-targeting tandem trimerbody (TT) fused to an engineered silent Fc region. This antibody (IgTT-4E1-S) was designed to combine the blockade of the PD-L1/PD-1 axis with conditional 4-1BB costimulation specifically confined to the tumor microenvironment (TME). The antibody demonstrated simultaneous binding to purified EGFR, PD-L1, and 4-1BB in solution, effective blockade of the PD-L1/PD1 interaction, and potent 4-1BB-mediated costimulation, but only in the presence of EGFR-expressing cells. These results demonstrate the feasibility of IgTT-4E1-S specifically blocking the PD-L1/PD-1 axis and inducing EGFR-conditional 4-1BB agonist activity.

## 1. Introduction

One of the most promising strategies for enhancing anti-tumor immune responses is the blockade of inhibitory immune checkpoints, such as cytotoxic T lymphocyte antigen 4 (CTLA-4), programmed cell death protein 1 (PD-1), or PD-1 ligand (PD-L1) [1]. Immune checkpoint blockers have transformed cancer treatment for a wide range of tumor types, but the overall response rates are still limited, as many patients have no response or only a transient response [2,3]. As of January 2024, seven PD-L1 blockers and eight PD-1 blockers have been approved for clinical use in the United States and Europe, with atezolizumab being the first anti-PD-L1 monoclonal antibody (mAb) on the market (2017) [4]. Another strategy involves targeting costimulatory pathways, such as 4-1BB, also known as CD137, a member of the TNF receptor (TNFR) superfamily (TNFRSF9), which is an activation-induced surface receptor that provides antigen-primed T cells with augmented survival, proliferation and effector functions, as well as metabolic advantages [5]. Anti-4-1BB agonistic mAbs have shown considerable potential in promoting tumor rejection in preclinical cancer models [6]. However, the clinical development of full-length anti-4-1BB antibodies has been hampered by off-tumor toxicity, which is mainly due to Fc-FcγR interactions [6,7,8]. Therefore, to fully exploit their therapeutic potential, novel approaches are being developed that generally aim to confine 4-1BB costimulation to the TME and draining lymph nodes by adding tumor-specific moieties to generate bispecific 4-1BB agonistic antibodies [8,9,10]. Tumor-associated antigens (TAAs), such as epidermal growth factor receptor (EGFR), fibroblast activation protein (FAP), CD19, B7-H3 (CD276), carcinoembryonic antigen (CEA), and EGFR 2 (HER2) have been targeted to develop 4-1BB bispecifics [8,9,10,11,12,13,14,15]. Recently, a range of bispecific constructs targeting 4-1BB-mediated T cell costimulation to PD-L1-overexpressing tumor cells and simultaneously blocking the PD-1/PD-L1 axis have been generated [16,17,18,19,20,21,22] and are being clinically evaluated.

Here, we generated and characterized a multispecific antibody by fusing a trispecific 4-1BB/EGFR/PD-L1 tandem trimerbody (TT) [23] with the human IgG_1_ hinge and Fc regions based on a previously described IgTT platform [24]. We used an engineered silenced Fc region to inhibit the binding of FcγR but retain the binding of FcRn for IgG-like pharmacokinetics [25]. The trispecific and hexavalent antibody was designed to simultaneously modulate two key pathways to enhance anti-tumor immune responses: PD-L1 blockade and tumor-specific 4-1BB costimulation. Our choice of TAA was the EGFR, a well-characterized tyrosine kinase receptor whose dysregulation promotes cancer cell proliferation, inhibits apoptosis, and promotes invasion [26].

## 2. Materials and Methods

### 2.1. Cell Lines and Culture Conditions

Dulbecco’s modified Eagle’s medium (DMEM) (Life Technologies, Carlsbad, CA, US; cat# 10313021) supplemented with antibiotics (100 units/mL of penicillin, 100 μg/mL of streptomycin; both from Life Technologies), 10% (*v*/*v*) heat-inactivated fetal bovine serum (FBS) (Merck Life Science, Darmstadt, Germany; cat# F7524-500 ML), and 2 mmol/L L-glutamine was used to culture HEK-293 (CRL-1573), NIH/3T3 (CRL-1658), CHO-K1 (CCL-61), and MDA-MB-231 (HTB-26) cells at 37 °C in 5% CO_2_. All these cell lines were obtained from the American Type Culture Collection. NIH/3T3 cells expressing human EGFR (3T3^EGFR^) were kindly provided by Dr. A. Villalobo (Instituto de Investigaciones Biomédicas “Alberto Sols”, IIBm CSIC-UAM, Madrid, Spain). Expi293F cells (from Gibco, Thermo Fisher Scientific, Waltham, MA, USA) were cultured in Expi293 expression medium in a humidified, 8% CO_2_ incubator rotating at 95 rpm at 37 °C. Jurkat T cells (TIB-152) were cultured in RPMI-1640 (Lonza Bioscience, Basel, Switzerland; cat# 12-702Q) supplemented with 10% (*v*/*v*) heat-inactivated FBS, 2 mmol/L of L-glutamine, and antibiotics. Jurkat T cells stably expressing human PD-1 and NFAT-induced luciferase (Jurkat^NFAT-PD-1^) and CHO-K1 cells stably expressing human PD-L1 (PD-L1 aAPC/CHO-K1) were obtained from Promega (Madison, WI, USA; cat# J1250). Jurkat T cells stably expressing human 4-1BB and NFAT-induced luciferase (Jurkat^NFAT-4-1BB^) were obtained from Promega (cat# JA2351). CHO-K1 cells stably expressing human PD-L1 (CHO^PD-L1^) were obtained from Genlantis (xCELLerateTM PD-L1 Stable Cell Line, XCL-PDL1), and CHO-K1 cells stably expressing human EGFR (CHO^EGFR^) were generated using human EGFR-encoding lentiviral particles (G&P Biosciences, Santa Clara, CA, US; cat# LTV0169). Jurkat T cells expressing GFP-tagged 4-1BB (Jurkat^4-1BB^) were generated by lentiviral transduction using commercial lentiviral particles (Origene, Rockville, MD, USA; cat# RC200664L4V). The ORF present in the lentivirus contains 4-1BB sequence followed by mGFP sequence in the C-terminus, a P2A cleavage site, and a puromycin resistance gene, all in frame. Briefly, 1 × 10^4^ cells were incubated with the lentiviral particles (titer of 5.5 × 10^7^ TU/mL) at a MOI (multiplicity of infection) of 20 for 8 h in retronectin-coated 24-well plates (20 µg/cm^2^). The cells were allowed to recover for 48 h and subsequently selected in 2 µg/mL of puromycin. The cells were maintained in culture in the presence of 1 µg/mL of puromycin. All cell lines were routinely screened for mycoplasma contamination by PCR using the Mycoplasma Plus TM Primer Set (Biotools B&M Labs, Madrid, Spain; cat# 90022).

### 2.2. Construction of the Expression Vectors

To generate the 4-1BB/EGFR/PD-L1 trispecific IgTT Fc^wt^ (IgTT-4E1) expression vector, the plasmid pCR3.1-FLAG/Strep-α4-1BB-αEGFR-αPD-L1-hFc^wt^-Myc-His was generated by cloning the insert OncoM-FLAG/Strep-α4-1BB scFv SAP3.28 [13] flanked by *Hind*III-*Not*I sites (GeneArt AG, Thermo Fischer, Waltham, MA, USA) and the insert αPD-L1 V_HH_ (Nb6p generated in llama immunized with human PD-L1 ectodomain [27]) flanked by *Afe*I-*Sac*II sites (GeneArt AG, Thermo Fischer) into the plasmid from pCR3.1-FLAG/Strep-αPD-L1-αEGFR-hFc^wt^-Myc-His encoding the bispefic IgTT-1E [24]. Then, to generate the 4-1BB/EGFR/PD-L1 trispecific IgTT Fc^silent^ (IgTT-4E1-S), the insert hFc^silent^-Myc-His flanked by *Sac*II-*Xba*I sites (GeneArt AG, Thermo Fischer) was subcloned into the plasmid pCR3.1-FLAG/Strep-α41BB-αEGFR-αPD-L1-hFc^wt^-Myc-His. Finally, to generate the 4-1BB/PD-L1/EGFR trispecific IgTT Fc^silent^ (IgTT-41E-S), the insert αPD-L1 V_HH_/αEGFR V_HH_ flanked by *Xho*I-*Sac*II sites (GeneArt AG, Thermo Fischer) was cloned into the plasmid pCR3.1-FLAG/Strep-α41BB-αEGFR-αPD-L1-hFc^silent^-Myc-His. All the sequences were verified using the FwCMV and RvBGH oligonucleotide primers (Table S1).

### 2.3. Expression and Purification of the Recombinant Antibodies

HEK-293 cells (4 × 10^5^ cells/well) were transfected with different vectors (2.5 µg of DNA/well) using a Lipofectamine 3000 kit (Fisher Scientific, Waltham, MA, USA; cat# 15292465) and cultured at 37 °C. The cells were then selected in complete DMEM supplemented with 500 μg/mL of G418 to generate stable cell lines. To produce large amounts of protein, 5.8 × 10^8^ Expi293F cells were transfected with 180 µg of DNA using the Expifectamine 293 transfection reagent (Life Technologies) in 180 mL of medium and cultured at 37 °C for four days. The conditioned media were collected and processed using the Strep-Tactin purification system (IBA Lifesciences, Göttingen, Germany) in an ÄKTA Prime plus system (Life Technologies). The purified antibodies were then dialyzed at 4 °C with PBS pH 7.4 supplemented with 150 mM NaCl, analyzed by sodium dodecyl sulphate (SDS)–polyacrylamide gel electrophoresis (PAGE) under reducing conditions, and assayed for endotoxin levels using a limulus amebocyte lysate (LAL) endotoxin quantitation kit according to the manufacturer’s specifications (Thermo Fisher Scientific, Waltham, MA, USA; cat# A39552S). The endotoxin levels were *<*0.25 EU/mL.

### 2.4. Enzyme-Linked Immunosorbent Assay

To determine the binding activity of the purified or secreted protein, human EGFR-Fc (hEGFR-Fc, R&D Systems, Minneapolis, MN, USA; cat# 344-ER), human PD-L1-Fc (hPD-L1-Fc, R&D Systems, cat# 156-B7-100), or human 4-1BB-Fc (h4-1BB-Fc, R&D Systems, cat# 838-4B-100) chimeras were immobilized (2.5 µg/mL in PBS) on Maxisorp 96-well plates (NUNC Brand Products, Thermo Fisher Scientific; cat# 44240) overnight at 4 °C. After washing and blocking with PBS–5% BSA, the conditioned medium or the purified protein solution (1 µg/mL) was added, and the plates were incubated for 1 h at room temperature. After washing, proteins were detected with HRP-conjugated anti-FLAG mAb (M2 clone, Sigma-Aldrich, Burlington, MA, USA; cat# A8592) (1 µg/mL) for 45 min at room temperature. The plates were then developed with 100 µL of 3,3′,5,5′-tetramethylbenzidine (TMB) (Sigma-Aldrich, cat# T0440), and the reaction was stopped with 100 µL of 1 N H_2_SO_4_. Absorbance was read at 450–620 nm using a Multiskan FC photometer (Thermo Scientific, Waltham, MA, USA).

### 2.5. Western Blotting

Conditioned medium samples containing 0.1% (*v*/*v*) FBS were analyzed by 10–20% tris-glycine SDS-PAGE under reducing conditions and transferred onto nitrocellulose membranes (Thermo Fisher Scientific, cat# IB23002). They were incubated with mouse anti-FLAG mAb (clone M2, Sigma-Aldrich, cat# F3165) (1 µg/mL) and probed with HRP-conjugated goat anti-mouse polyclonal antibody (GAM-HRP) (Sigma-Aldrich, Burlington, MA, USA; cat# A2554) (1:10,000 dilution). The visualization of protein bands was performed with Pierce ECL Plus Western Blotting Substrate (Thermo Scientific, cat# 32132) using the ChemiDoc MP Imaging System and Image Lab software (version 6.0.1, both from BioRad, Hercules, CA, USA).

### 2.6. Size-Exclusion Chromatography

Size-exclusion chromatography (SEC) was performed on a Superdex 200 Increase 10/300 GL column (Cytiva) on an ÄKTA go chromatography system (Cytiva). The column was equilibrated in 0.22 μm filtered PBS. Analytical runs of the antibody were performed at room temperature at a flow rate of 0.75 mL/min, injecting 20 µg of protein. The column was calibrated using the Gel Filtration HMW Calibration Kit from Cytiva (cat# 28403842). Data acquisition and analysis were performed using ASTRA software (version 7.6, Wyatt Technology, Santa Barbara, CA, USA). The reported molar mass corresponds to the center of the chromatography peaks.

### 2.7. Molecular Modeling

The three-dimensional representation of IgTT-4E1 was built by comparative homology modeling using MODELLER [28]. The model was created by using different templates for the different domains. The V_HH_ monospecific TT model was generated utilizing as a template the monospecific anti-CEA TT from a prior study [23]. The Fc region was modeled based on the human IgG_1_ B12 structure (pdb:1HZH.H) [29], obtained from the Protein Data Bank [30] (99% sequence identity, e-value of 1 × 10^−169^ as calculated by BLAST (version 2.2.24) [31]). The 1HZH structure also guided the dimer modeling. The scFv domain was modeled using the interleukin 18 receptor antagonist scFv (pdb:6NK9.D) [32] as a template (83% sequence identity, e-value of 2 × 10^−138^ as calculated by BLAST). Ultimately, the final representation was generated by merging the models.

### 2.8. Biolayer Interferometry

The simultaneous binding of all three cognate antigens by IgTT-4E1 was investigated using biolayer interferometry (BLI) on an Octet RED96 system (Fortebio, Fremont, CA, USA). We immobilized 5 g/L of hEGFR-Fc onto AR2G biosensors (Fortebio) at pH 5.0 using amine-reactive coupling. Then, IgTT-4E1 in HEPES-buffered saline (HBS; 20 mM HEPES, 150 mM NaCl, pH 7.4) at 20 nM was allowed to associate with the hEGFR-Fc-coated biosensors for 20 min, after which the dissociation of the antibody from the biosensors was measured for 5 min in HBS buffer only. The biosensors were then moved into HBS containing 50 nM hPD-L1-Fc for 20 min and subsequently into HBS containing 50 nM h4-1BB-Fc for 20 min, followed by 5 min of dissociation in HBS.

### 2.9. Flow Cytometry

The purified antibody IgTT-4E1 or control monospecific mAbs (6.67 nM) were incubated with CHO, CHO^EGFR^, CHO^PD-L1^, 3T3, 3T3^EGFR^, Jurkat, or Jurkat^4-1BB^ T cells (1 × 10^5^ cells/well) for 30 min on ice. After washing, PE-conjugated F(ab’)_2_ goat anti-human (GAH) polyclonal antibody (Jackson ImmunoResearch, West Grove, PA, USA; cat# 109-116-170) was added and incubated for 30 min. Rituximab (Fritz Hoffmann-La Roche, Basel, Switzerland), urelumab (MedChemExpress, Monmouth Junction, NJ, USA; cat# HY-p99055), atezolizumab (Fritz Hoffmann-La Roche), and cetuximab (Merck KGaA, Darmstadt, Germany) (6.67 nM) were used as monospecific controls. To immunophenotype MDA-MB-231 cells and peripheral blood mononuclear cells (PBMC), the following mAbs were used: PE-conjugated anti-EGFR (Becton Dickinson, Franklin Lakes, NJ, USA; cat# 555997), APC-conjugated anti-PD-L1 (BD, cat# 563741), FITC-conjugated anti-PD-1 (BD, cat# 557860), PE-conjugated anti-CD137 (BD, cat# 555956), PE-conjugated isotype IgG_1_ (BD, cat# 554680), APC-conjugated isotype IgG_1_ (BD, cat# 554681), and FITC-conjugated isotype IgG_1_ (ImmunoTools; Friesoythe, Germany; cat# 21275513) and incubated for 30 min. Finally, after washing, DAPI (Sigma Aldrich, cat# D9542) was added, and the samples were analyzed with a FACSCanto II flow cytometer (Becton Dickinson).

### 2.10. ADCC Reporter Bioassay

The ADCC reporter bioassay (Promega, cat# G7010) was performed according to the manufacturer’s instructions. Briefly, 3T3 or 3T3^EGFR^ cells (1.2 × 10^4^/well) were seeded in 96-well white plates in DMEM-10% FBS and incubated overnight at 37 °C. Then, the medium was removed, and different amounts of rituximab, IgTT-4E1, or IgTT-4E1-S (final concentrations 66.7; 6.67; 0.667 nM) were added in 40 µL of RPMI-1% FBS/well. Then, Jurkat^NFAT-CD16^ effector cells (7.5 × 10^4^/well) were added to RPMI-1% FBS (40 µL/well) and incubated for 6 h at 37 °C. Finally, 80 µL/well of BioGlo Reagent (Promega) was added, and bioluminescence was measured in a Tecan Infinite F200 Fluorescence Microplate Reader (Life Sciences, St. Petersburg, FL, USA; Tecan).

### 2.11. PD-1/PD-L1 Blockade Bioassay

The PD-1/PD-L1 Bioassay (Promega, cat# J1250) was used following the manufacturer’s instructions. Briefly, PD-L1 aAPC/CHO-K1 cells (2.5 × 10^4^/well) were seeded in 96-well white plates in DMEM-10% FBS and incubated overnight at 37 °C. Then, the medium was removed, and different amounts of atezolizumab, cetuximab, or IgTT-4E1-S (final concentrations 66.7; 6.67; 0.667 nM) were added in RPMI-1% FBS (40 µL well). Then, Jurkat^NFAT-PD-1^ cells (1.25 × 10^5^/well) were added to RPMI-1% FBS (40 µL/well) and incubated for 6 h at 37 °C. Finally, 80 µL/well of BioGlo Reagent was added, and bioluminescence was measured in a Tecan Infinite F200 Fluorescence Microplate Reader.

### 2.12. Antigen-Dependent Jurkat 4-1BB Activation Assay

The 4-1BB Bioassay (Promega, cat#. J2332) was used, following the manufacturer´s instructions. Briefly, CHO, CHO^EGFR^, or CHO^PD-L1^ cells (3 × 10^4^/well) were seeded in 96-well white plates in DMEM-10% FBS and incubated overnight at 37 °C. Then, the medium was removed, and different amounts of urelumab, rituximab, purified IgTT-4E1-S (final concentrations 66.7; 6.67; 0.667 and 0.0667 nM), or conditioned media containing IgTT-1E, IgTT-4E1-S, and IgTT-41E-S were added. Then, Jurkat^NFkB-4-1BB^ cells (1.25 × 10^5^/well) were added to RPMI-1% FBS (40 µL/well) and incubated for 6 h at 37 °C. Finally, 80 µL/well of BioGlo Reagent was added, and bioluminescence was measured in a Tecan Infinite F200 Fluorescence Microplate Reader.

### 2.13. IFNγ Secretion Analysis

Human peripheral blood mononuclear cells (PBMCs) were isolated from healthy donors by density gradient centrifugation (Lymphoprep, Axis–Shield, Dundee, UK; cat# AXS-1114544). All donors provided a written informed consent in accordance with the Declaration of Helsinki. PBMCs were activated overnight with 0.3 μg/mL of anti-CD3 mAb (OKT3; BD, cat#. 566685) and co-cultured with MDA-MB-231 cells (5 × 10^4^/well) at an effector/target (E/T) ratio of 1:1 (5 × 10^4^ cells/well) and 2:1 (1 × 10^5^ cells/well) in the presence of 6.67 nM control polyclonal human IgG (Griffols, Barcelona, Spain), urelumab, atezolizumab, or IgTT-4E1-S for 48 h. The supernatants were collected, and IFNγ levels were analyzed by ELISA (Bionova, Fremont, CA, USA; cat# 851.560.010) following the manufacturer’s instructions. The experiment was performed in triplicate.

### 2.14. Statistical Analysis

All graphs and statistical analyses were performed using GraphPad Prism 9.0. Most in vitro experiments were performed in triplicate, and the values are presented as mean ± SD. Significant differences (*p* value) were identified using one-way analysis of variance (ANOVA), adjusted by Dunnett’s test for multiple comparisons, as indicated. *p* values are shown in the corresponding figures. Two-way ANOVA was used to analyze experiments that evaluated the interaction of two variables, such as cell type and therapy, following multiple comparison testing using either Dunnett’s or Tukey’s test, as appropriate.

## 3. Results

### 3.1. Generation of IgTT-4E1 and IgTT-4E1-S

**Figure 1:**
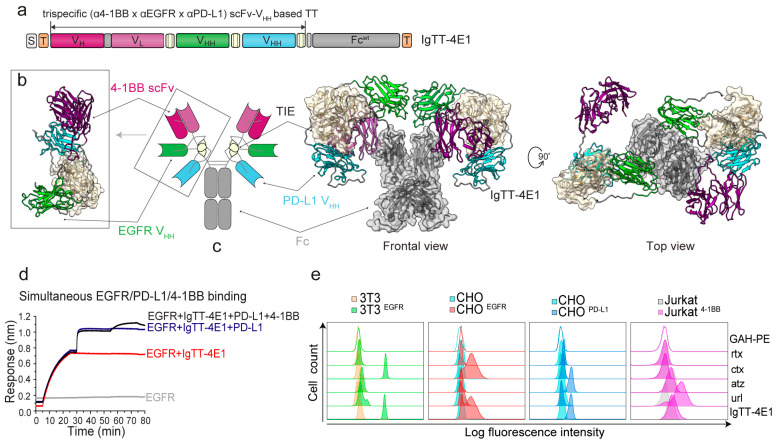
Gene construct, molecular model, and binding characteristics of the IgTT-4E1 antibody. (**a**) Gene layout of the IgTT-4E1 antibody bearing a signal peptide from oncostatin M (white box), one anti-4-1BB scFv (magenta boxes), one anti-EGFR V_HH_ (green box), one anti-PD-L1 V_HH_ (blue box), three collagen-derived trimerization (TIE) domains (yellow boxes) flanked by peptide linkers, and the Fc-encoding element (gray box). N-terminal FLAG-Strep and C-terminal Myc-His tags (orange boxes) were appended for purification and immunodetection purposes. (**b**) Schematic diagram showing the three-dimensional model of the anti-4-1BB/EGFR/PD-L1 tandem trimerbody (TT). (**c**) Molecular diagram of the IgTT-4E1 antibody and its three-dimensional modelizations, in front and top views. (**d**) Human EGFR-Fc (EGFR) was immobilized onto four different biosensors; three biosensors were incubated in 20 nM IgTT-4E1 for 20 min (red trace), while the fourth was kept in HBS as a negative control (gray trace). Then, 50 nM human PD-L1-Fc (PD-L1) was added to two of the IgTT-4E1-loaded biosensors (blue trace) and the control biosensor for 20 min. Also, 50 nM human 4-1BB-hFc (4-1BB) was then added to one PD-L1- and IgTT-4E1-treated biosensor (black trace) and the control biosensor. The cumulative signal increases on the biosensor receiving IgTT-4E1, PD-L1, and 4-1BB demonstrate that all three antigens can be bound by the IgTT-4E1 antibody simultaneously. (**e**) The binding to human EGFR, PD-L1, and 4-1BB on the cell surface of 3T3, 3T3^EGFR^, CHO, CHO^EGFR^, CHO^PD-L1^, Jurkat, and Jurkat^4-1BB^ cells by rituximab (rtx), cetuximab (ctx), atezolizumab (atz), urelumab (url), and IgTT-4E1 at 6.67 nM was measured by FACS. Cells incubated with PE-conjugated GAH antibody (GAH-PE) are shown as non-filled histogram. The *y*-axis shows the relative cell number, and the *x*-axis represents the intensity of fluorescence expressed on a logarithmic scale.

In this study, we generated a trispecific 4-1BBxEGFRxPD-L1 IgTT by replacing the N-terminal scFv from IgTT-1E [24] with an anti-4-1BB agonistic scFv [13], and the C-terminal anti-EGFR V_HH_ with an anti-PD-L1 blocking V_HH_ [27] (IgTT-4E1, Figure 1a). This antibody was further modified by replacing the parental wild-type IgG_1_ Fc region with an engineered Fc region containing P329G L234A/L235A (PGLALA) mutations attenuating FcγR binding [33] (IgTT-4E1-S, Figure S1a). As shown in Figure 1b,c, the middle V_HH_ (green) and the C-terminal V_HH_ (blue) were each connected to the trimerization domains by two linkers, whereas the N-terminal scFv (magenta) had a single linker, providing the binding domains with variable distances and broad areas of action around the central Fc region. Both IgTT-4E1 and IgTT-4E1-S antibodies were purified from the conditioned medium of transfected Expi293 cells by Strep-Tactin affinity chromatography and eluted as a single and an symmetrical peak with protein yields of approximately 13 mg/L. The analysis of the purified IgTT-4E1-S by SEC showed a major symmetric peak with an elution volume corresponding to an experimental molecular mass of 305 kDa, in accordance with the calculated value for the dimeric species in solution (240 kDa) (Figure S1b). A major band corresponding to the IgTT-4E1-S monomer was identified by visual inspection of the reducing SDS-PAGE analysis after SEC (Figure S1c). Both IgTT-4E1 and IgTT-4E1-S specifically recognized human 4-1BB-Fc (4-1BB), human EGFR-Fc (EGFR), and human PD-L1 Fc (PD-L1) (Figure S1d).

BLI experiments were used to investigate whether IgTT-4E1 could simultaneously bind to all three antigens. First, IgTT-4E1 was found to interact with EGFR immobilized onto biosensors. Then, the IgTT-4E1-loaded biosensors were incubated with PD-L1 and subsequently with 4-1BB, which resulted in cumulative increases in the signal (Figure 1d). The signal increases were not observed when the antigens were incubated with EGFR-coated biosensors not loaded with IgTT-4E1, demonstrating that they in fact represented complex formation with IgTT-4E1 and not non-specific interactions with EGFR or the biosensor surface. Complex formation between IgTT-4E1 and all three antigens showed that IgTT-4E1 is functionally trispecific, demonstrating a lack of steric hindrance between its three cognate interactions in this experimental context.

The ability to specifically detect antigens in a cellular context was studied by flow cytometry, using urelumab, cetuximab, and atezolizumab as binding controls, and rituximab as a negative control (Figure 1e). With Jurkat^4-1BB^ cells, a specific displacement in fluorescence intensity was observed with IgTT-4E1 and urelumab compared to untransfected Jurkat cells. A specific increase in fluorescence intensity was observed upon the addition of both IgTT-4E1 and cetuximab to EGFR-positive cells (3T3^EGFR^ and CHO^EGFR^) when compared to EGFR-negative cells (3T3 and CHO). For CHO^PD-L1^ cells, a shift is shown with IgTT-4E1 and atezolizumab, with respect to CHO cells. Moreover, with IgTT-4E1 and atezolizumab, a shift was observed in both Jurkat and Jurkat^4-1BB^ cells with respect to the control, due to PD-L1 expression in Jurkat cells.

### 3.2. Determination of Fc-Mediated Effector Functions

**Figure 2:**
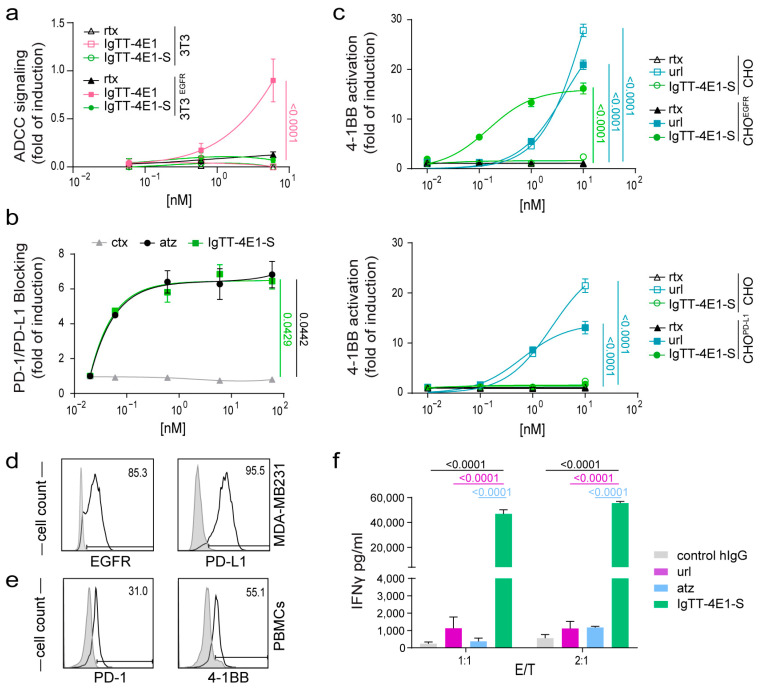
Effect of IgTT-4E1-S on PD-L1/PD-1 blockade, 4-1BB costimulation, and IFNγ secretion. (**a**) ADCC reporter bioassay response to rituximab (rtx), IgTT-4E1, and IgTT-4E1-S, using ADCC bioassay effector Jurkat^NFAT-CD16^ cells co-cultured with 3T3 or 3T3^EGFR^ target cells at a 6:1 E/T ratio for 6 h at 37 °C. After incubation, Bio-Glo™ Luciferase Assay Reagent was added for luminescence determination. Data shown represent the mean ± standard deviation of triplicates. Data are presented as the mean ± SD (*n* = 3). Significance was determined by two-way ANOVA with Tukey’s multiple comparison test. (**b**) PD-L1/PD-1 blockade bioassay assesses the inhibitory activities of IgTT-4E1-S. *Y*-axis represents reporter gene fold induction. Cetuximab (ctx) was used as a negative control, and atezolizumab (atz) as a positive control. Fold induction relative to negative control (ctx)-incubated cells. Results are expressed as mean ± SD (*n* = 3). Significance was measured by one-way ANOVA with Dunnett’s multiple comparison test. (**c**) Costimulation of Jurkat^NFkB-4-1BB^ cells co-cultured with CHO cells, CHO^EGFR^, or CHO^PD-L1^ cells in the presence of 10-fold increasing concentrations of IgTT-4E1-S, urelumab (url), and rituximab (rtx) antibodies for 6 h at 37 °C. After incubation, luminescence was determined. Data are presented as fold induction relative to the values obtained from unstimulated Jurkat^NFkB-4-1BB^ cells. Representative dose–concentration curves are presented and expressed as mean ± SD (*n* = 3). Significance was determined by two-way ANOVA with Tukey’s multiple comparison test. (**d**) Flow cytometry analysis of EGFR and PD-L1 expression in MDA-MB-231 cancer cells. Cells incubated with PE-conjugated and APC-conjugated isotypes are shown as gray-filled histogram. (**e**) Flow cytometry analysis of PD-1 and 4-1BB expression in PBMCs preactivated with anti-CD3 mAb for 24 h. PBMCs incubated with FITC-conjugated and PE-conjugated isotypes are shown as gray-filled histogram. (**f**) Anti-CD3 preactivated PBMCs were co-cultured with MDA-MB231 target cells at effector/target (E/T) ratios of 1:1 and 2:1. Control human polyclonal IgG (control hIgG), urelumab (url), atezolizumab (atz), and IgTT-4E1-S were added at 6.67 nM. The IFNγ concentrations in supernatant after 48 h were analyzed and expressed as mean ± SD (*n* = 3). Significance was determined by two-way ANOVA with Tukey’s multiple comparison test.

To measure differences in ADCC activity, Jurkat cells constitutively expressing human FcγRIIIa (CD16) on the cell surface (Jurkat^NFAT-CD16^) and a luciferase reporter driven by an NFAT response element were co-cultured with 3T3^EGFR^ cells or with 3T3 cells as a negative control. IgTT-4E1 induced the activation of Jurkat^NFAT-CD16^ cells in co-cultures with 3T3^EGFR^ cells, as evidenced by a significant increase in luciferase activity (*p* < 0.0001; Figure 2a), while the IgTT-4E1-S protein and rituximab (negative control) did not show an increase in luciferase activity. In the absence of EGFR-mediated interactions (co-cultures with 3T3 cells), rituximab and both IgTT-4E1 and IgTT-4E1-S showed no induction on Jurkat^CD16^ cells.

### 3.3. Effect of IgTT-4E1-S on PD-1/PD-L1 Blockade

To demonstrate the ability of IgTT-4E1-S to block PD-1/PD-L1 interactions, Jurkat cells expressing human PD-1 and a luciferase reporter driven by an NFAT response element (Jurkat^NFAT-PD-1^) were co-cultured with APC/CHO-K1 cells, which express human PD-L1, and an engineered cell surface protein designed to activate cognate TCRs in an antigen-independent manner. As shown in Figure 2b, IgTT-4E1-S efficiently blocked PD-1/PD-L1 interactions, leading to a significant induction of luciferase activity in Jurkat^NFAT-PD-1^ cells (*p* = 0.0429), similar to that observed with the PD-L1 blocking antibody atezolizumab (*p* = 0.0442). In contrast, no PD-1/PD-L1 blocking activity was observed in the presence of cetuximab.

### 3.4. Costimulatory Activity of IgTT-4E1-S

The agonist activities of IgTT-4E1-S and urelumab were assessed using Jurkat cells stably transfected with NFκB-inducible luciferase and 4-1BB (Jurkat^NFkB-4-1BB^), co-cultured with target cells expressing either EGFR (CHO^EGFR^) or PD-L1 (CHO^PD-L1^), and untransfected CHO cells as a negative control. In the absence of EGFR- or PD-L1-mediated cross-linking at the target cell surface (CHO cells), IgTT-4E1-S showed no induction in untreated Jurkat^NFkB-4-1BB^ cells at all tested concentrations, whereas urelumab showed an approximately 25-fold induction (EC_50_ = 0.3115 nM, Figure 2c). In the presence of EGFR-mediated cross-linking (i.e., using CHO^EGFR^ as target cells), IgTT-4E1-S induced an NFκB dose-dependent activation with a 15-fold induction (*p* < 0.0001). Effective costimulation was achieved at concentrations that were an order of magnitude lower than those of urelumab (EC_50_ = 0.02635 nM) (Figure 2c). In contrast, in co-cultures with PD-L1-positive cells (CHO^PD-L1^), IgTT-4E1-S showed no induction of luciferase at any concentration tested (Figure 2d). The negative control rituximab showed no activation (Figure 2c,d).

Thus, IgTT-4E1-S demonstrated potent and conditional costimulation that was dependent on EGFR but not on PD-L1 expression. To further investigate this issue, we generated an additional IgTT silent molecule with the order of the V_HH_ domains reversed (Figure S1a). This construct (IgTT-41E-S) was also efficiently secreted, and Western blot analysis under reducing conditions showed a migration pattern consistent with the molecular weight calculated from their amino acid sequence (Figure S2a). ELISA analysis demonstrated that IgTT-41E-S specifically recognized 4-1BB, EGFR, and PD-L1 (Figure S2b). In the presence of PD-L1-expressing cells, IgTT-41E-S also showed no induction of luciferase in Jurkat^NFkB-4-1BB^ cells. However, in the presence of EGFR cells, IgTT-41E-S showed a fold induction similar to that observed with IgTT-4E1-S (Figure S2c). These results show that EGFR but not PD-L1 enabled cross-linking-mediated 4-1BB signaling with two different configurations of the PD-L1- and EGFR-binding V_HHs_, indicating that the issue did not arise from steric hindrance of the PD-L1-binding domain.

### 3.5. IgTT-4E1-S Enhances the Activation of Primary Human T Cells

The triple-negative breast cancer MDA-MB-231 cells expressing both EGFR and PD-L1 (Figure 2d) were co-cultured for 48 h at two different effector/target ratios (1:1 and 2:1) with anti-CD3-prestimulated PBMCs expressing PD-1 and 4-1BB (Figure 2e). The agonistic anti-4-1BB mAb (urelumab) and anti-PD-L1 blocking mAb (atezolizumab) had a modest effect on IFNγ production (Figure 2f). In contrast, the IgTT-4E1-S antibody strongly enhanced IFNγ secretion levels, which were significantly higher (*p* < 0.0001) than those observed in the single-treated co-cultures (Figure 2f).

## 4. Discussion

The design of multi-specific antibodies against a combination of immunomodulatory targets is a promising approach to enhance the clinical benefit of conventional checkpoint blockers. Here, we generated a trispecific IgG-like antibody by fusing an anti-4-1BB x anti-EGFR x anti-PD-L1 TT with an engineered Fc silent region to abrogate FcγR binding. This molecule was based on the previously described IgTT platform, which is a fusion of a mono- or multispecific TT with an Fc region to generate a hexavalent IgG-like antibody capable of bivalently recognizing up to three different antigens [24]. The binding domains of IgTT-4E1 are positioned around the human collagen homodimerization domain, and BLI studies showed that IgTT-4E1 could bind all three antigens simultaneously in solution. Furthermore, IgTT-4E1 was able to recognize the antigens in a cellular context. These results suggest that the binding domains are predominantly sterically unhindered and available for antigen binding.

Anti-4-1BB agonistic mAbs can be classified as either strong or weak agonists. Here, we confirmed in a 4-1BB-reporting cell line that urelumab, a strong agonist, could induce signaling activation without FcγR-mediated cross-linking. The anti-4-1BB antibody used to generate IgTT-4E1-S is a weak agonist [13], and therefore 4-1BB signaling was only induced with additional cross-linking. IgTT-4E1-S exhibited conditional 4-1BB agonist activity dependent on the cross-linking with EGFR but not with PD-L1. This finding was independent of the position of the anti-EGFR and anti-PD-L1 V_HH_ domains in the TT, suggesting that the agonist activity was not influenced by steric hindrance issues. These results are not consistent with those published for other anti-4-1BB × anti-PD-L1 antibodies [19,20,21,22,34,35], where conditional 4-1BB agonist activity dependent on PD-L1 cross-linking was reported. These differences in the ability of IgTT to induce 4-1BB-mediated costimulation may be related to structural determinants or to the epitopes recognized by the binding domains. The anti-4-1BB antibody binds to the N-terminal CRD1 domain [36]; anti-EGFR V_HH_ binds in the cleft formed between domains II and III [37], while the epitope recognized by anti-PD-L1 V_HH_ has not been characterized. In the IgTT molecule, each TT is trispecific, and the three binding domains are very close and spatially restricted, which may limit the potential for the simultaneous recognition of distally located epitopes and epitopes more proximal to the membrane [38], as might occur with 4-1BB (86 kDa) and PD-L1 (30 kDa) interactions. This would probably not be a limitation with conventional bispecific antibodies, where each arm recognizes a different antigen, and the area of influence is significantly greater. In the context of simultaneous interactions between 4-1BB and EGFR (134 kDa) mediated by IgTT-4E1-S, available structural data suggest that the epitopes may be in closer proximity to each other. The ability to block the PD-L1/PD-1 interaction in a CHO cell system was similar to that obtained with atezolizumab. This was also previously observed with a dimeric version of the same anti-PD-L1 V_HH_ by using an ELISA-type blocking assay [27]. Simultaneous targeting of PD-L1 and EGFR by bispecific antibodies promotes PD-L1 blockade selectively in the TME due to EGFR overexpression in malignant cells, while reducing potential off-tumor binding to normal PD-L1-expressing cells [35]. In addition, EGFR overexpression and activation enhances PD-L1 expression by tumor cells [39]. IgTT-4E1-S enhanced the activation and effector functions of human primary T cells co-cultured with EGFR^+^/PD-L1^+^ cancer cells. However, to determine the ability of IgTT-4E1-S to restrict 4-1BB costimulation to EGFR-expressing tumors while minimizing off-target costimulation, further studies are required in cancer cell lines expressing different levels of EGFR and PD-L1, as well as in humanized mouse models carrying patient-derived xenografts. In addition, IgTT-4E1-S carries a silenced Fc domain to avoid or minimize anti-4–1BB-induced hepatic damage, which is partly explained by Fc-Fc*γ*Rs interactions [6,7,8], but studies in non-human primates are warranted to further explore its toxicity profile.

In summary, here we described the generation of IgTT-4E1-S, the first IgG-like trispecific antibody designed to provide EGFR-conditional 4-1BB stimulation to antigen-experienced T cells while constitutively blocking the PD-1/PD-L1 inhibitory axis. This apparent asymmetry between conditional 4-1BB agonism and PD-L1 blockade may be relevant when comparing our antibody to other bispecific antibodies already on the market [17,20,22,35], but further studies are needed to determine the efficacy and safety of the IgTT-4E1-S antibody.

## Data Availability

The datasets used and/or analyzed during the current study are available from the corresponding author [L.Á.-V.] on reasonable request.

## Supplementary Materials

The following supporting information can be downloaded at: https://www.mdpi.com/article/10.3390/antib13020034/s1, Figure S1: Gene constructs and structural characterization of the IgTT-4E1-S antibody; Figure S2: Characterization of the IgTT-41E-S antibody; Table S1: Oligonucleotides used in this study.
