# Supplementary material for: Characterization of a Trispecific PD-L1 Blocking Antibody That Exhibits EGFR-Conditional 4-1BB Agonist Activity
Source: Antibodies (Basel). 2024 Apr 24;13(2):34. doi: 10.3390/antib13020034 (PMC11130918; doi:10.3390/antib13020034)
Supplement: Supplementary file 1 [file antibodies-13-00034-s001.zip › antibodies-2846496-supplementary.pdf]

Supplementary materials

Characterization of a Trispecific PD-L1 Blocking Antibody That Exhibits EGFR-Conditional 4-1BB Agonist Activity

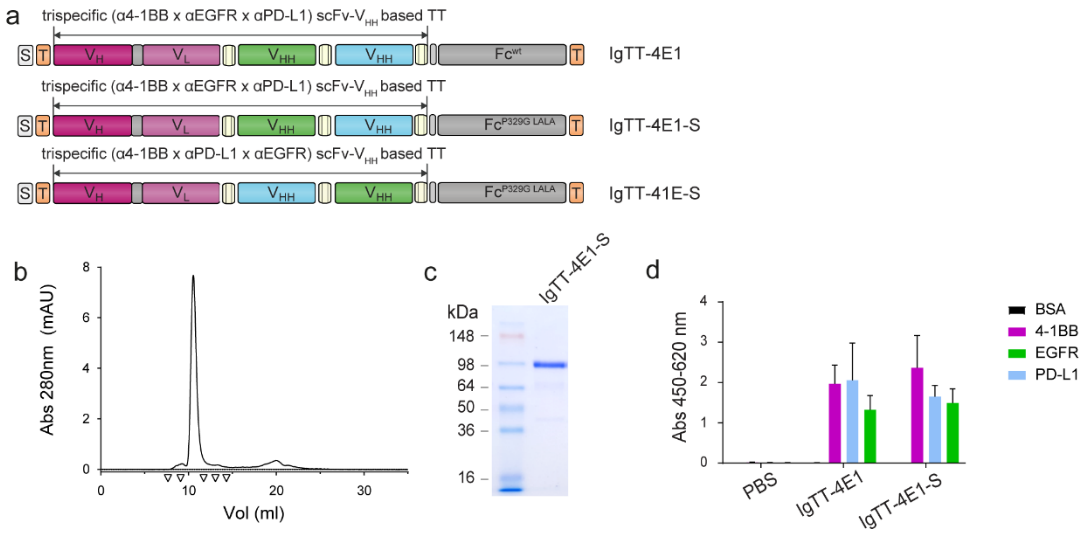

Figure S1. Gene constructs and structural characterization of IgTT-4E1-S antibody. (a) Gene layout of the IgTT-4E1, IgTT-4E1-S and IgTT-41E-S bearing a signal peptide from oncostatin M (white box), one anti-4-1BB scFv (magenta boxes), one anti-EGFR V_{HH} (green box), one anti-PD-L1 V_{HH} (blue box), three collagen-derived trimerization (TIE) domains (yellow boxes) flanked by peptide linkers and the Fc encoding element (gray boxes). N-terminal FLAG-Strep and C-terminal Myc-His tags (orange boxes) were appended for purification and immunodetection purposes. **(b)** SEC analysis of IgTT-4E1-S molecules in Superdex 200 Increase 10/300 GL column. Experiments were performed once after calibrating the column using a Gel Filtration HMW Calibration Kit from Cytiva**.** ▽ Corresponds to the elution volumes of the well-defined protein standards used for calibrating, from left to right: thyroglobulin (8.73ml), ferritin (9.89 ml), aldolase (12.11 ml), conalbumin (13.59 ml) and ovalbumin (14.60 ml). **(c)** Reducing SDS-PAGE analysis of the central fraction of the major peak from SEC experiment. **(d)** ELISA analysis where both purified protein IgTT-4E1 and IgTT-4E1-S specifically recognized human 4-1BB-Fc (4-1BB), human EGFR-Fc (EGFR) and human PD-L1- Fc (PD-L1) followed by anti-FLAG-HRP detection.

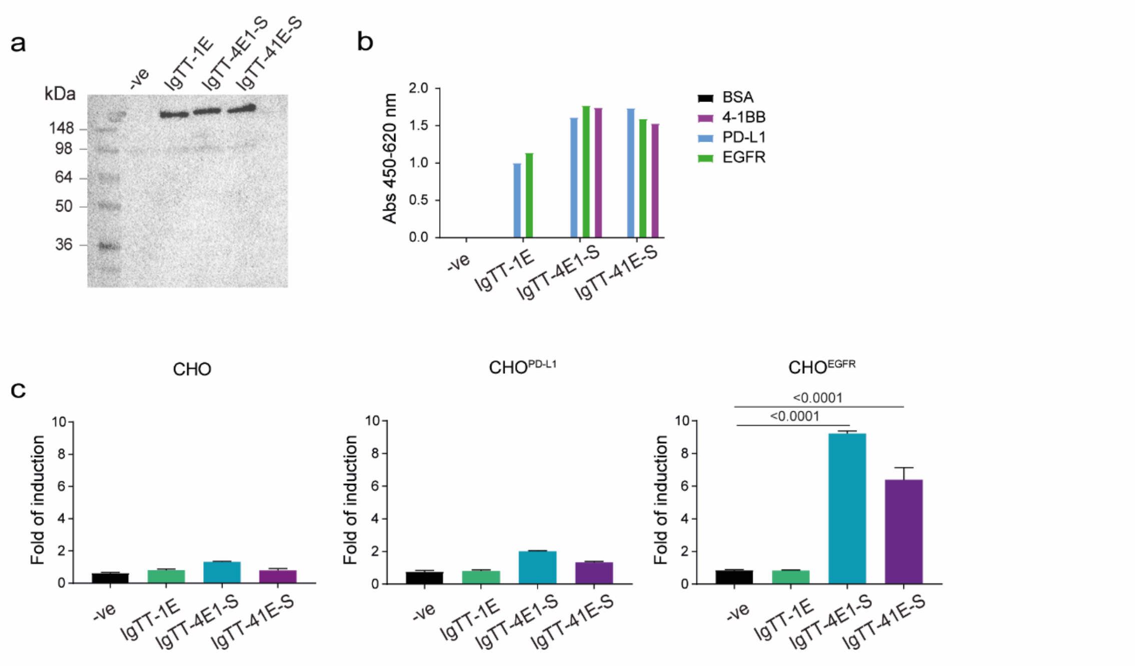

Figure S2. Characterization of IgTT-41E-S antibody. (a) Conditioned media (0.1% FBS) from negative control (vehicle), IgTT-1E, IgTT-4E1-S and IgTT-41E-S were analyzed by Western blot and probed with mouse anti-FLAG IgG1, followed by incubation with GAM-HRP. Three proteins were efficiently secreted by Expi293 cells and exhibited a migration pattern consistent with the molecular weight calculated from the amino acid sequences. **(b)** ELISA analysis where three purified proteins specifically recognized ELISA analysis where both purified protein IgTT-4E1 and IgTT-4E1-S specifically recognized human 4-1BB-Fc (4-1BB), human EGFR-Fc (EGFR) and human PD-L1- Fc (PD-L1) followed by anti-FLAG-HRP detection. **(c)** T cell co-stimulation of Jurkat$^{NFkB-4-1BB}$ cells co-cultured with CHO cells, CHO^{EGFR} or CHO^{PD-L1} cells in the presence of conditioned media of negative control (vehicle), IgTT-1E, IgTT-4E1-S and IgTT-41E-S, and after 6 hours at 37 °C luminescence determined. Data were presented as fold induction relative to the values obtained from unstimulated Jurkat$^{NFkB-4-1BB}$ cells. Results are expressed as mean ± SD (n = 3). Significance was measured by one-way ANOVA with Dunnett's multiple comparison test.

Table S1. Oligonucleotides used in this study

**Name**	**Sequence (5´-3´)**
FwCMV	CGCAAATGGGCGGTAGGCGTG
RvBGH	TAGAAGGCACAGTCGAGG

Oligonucleotides were synthesized by Thermo Scientific.
